# Oxygen—Its Agency in Therapeutics

**Published:** 1887-02

**Authors:** B. M. Lawrence


### OXYGEN, ITS AGENCY IN THERAPEUTICS.

BY B. M. LAWRENCE, M. D.

### No. 3.

There is no occupation or position in life more important or responsible than that of a physician. From the hour of birth until the time of death the physical welfare of every member of society depends more or less upon the doctor's skill. The profession has no place for the selfish or narrow-minded, and every member of the various schools of medicine who is worthy of his calling should be practically Eclectic, or as the term implies, he should be ever ready and willing to use any remedy or method of cure—old or new; regular or irregular—providing both reason and experience clearly prove it to be the best, safest and most successful. The man or woman with only one idea should never try to connect that idea, however brilliant, with the art of healing the sick. Looking back for a generation past, we find that many most marvelous inventions and improvements have been made. In every department of life, new and wonderful discoveries have followed each other in rapid succession. The fairy tales of the olden times appear insignificant when compared with the scientific achievements of the nineteenth century, when the mighty earth on every land is threaded with a nervous net work of wires, to the ends of which we whisper our anxious thoughts, and with the speed of light, our loves, our hopes or fears are repeated in the listening ear a thousand miles in the distance.

But, the question arises : Has the science of medicine kept pace with the grand march of progress which characterizes the wonderful age in which we are living ?

While men are ever willing to sacrifice their lives for principle, for love, or honor, while the soldier stands ready to "sack the bubble reputation at the cannon's mouth," is the doctor as willing, if needs be, to enter death's pale realm to find one germ of truth, destined, when brought to light, to benefit and bless mankind ? The true physician accepts the truth wherever found; he gladly interrogates the oracles of nature and strives if possible to penetrate the unseen world of thought and bring its hidden treasures forth to preserve and protect the priceless boon of health. Every day the evidence of medical progress becomes more apparent, both with the people and with physicians. HEALTH JOURNAL subscriptions are constantly increasing, and medical books are being rapidly multiplied. Many new and more rational remedies are being introduced, while a large class of the community dispense, as far as possible, with all forms of drugs, and trust more to a careful observance of the laws and conditions of health.

Progressive physicians, in all parts of the country, are more ready to investigate new theories, and to accept any rational improvement. These facts are sufficient to account for the rapid increase, in all parts of the country, among physicians of every school, of the Oxygen Treatment, or the use of oxygen, minoxide of nitrogen, and ozone compounds in the form of gasses medicated and employed by means of inhalation direct to the lungs, blood, and thence to the nervous system. Drs. Starkey and Palen, of Philadelphia, have probably been more instrumental in forcing the claims of oxygen compounds upon both the profession and the people than all other means combined. Through the influence of judicious, well-conducted advertising in all the first-class journals, and by their own publications, they were years since successful in receiving the patronage of many most distinguished men and women, occupying highly responsible positions in all the walks of life, social, political and religious. The following testimonials are samples of thousands which have been freely offered for publication.

HON. WILLIAM D. KELLEY wrote the following concerning his experience with the Oxygen Treatment:

WEST PHILADELPHIA, June 6th, 1877.

"DR. G. R. STARKEY, Philadelphia:

"*Dear Sir:*—Just about four years have elapsed since, overcoming a violent prejudice against any treatment that was offered as a specific for a wide range of apparently unrelated diseases, I yielded to the wishes of my friends, and, abandoning other medicine, put myself in your charge.

"Gratitude to you and duty to those who may be *suffering as I was from chronic catarrh* and almost daily effusion of blood, in greater or less quantities, but always sufficient to keep one reminded of his mortality, impel me to say to you and *to authorize you to give any degree of publicity to my assertion*, that the use of your gas, at intervals, has so far restored my health that I am not conscious of having discharged any blood for more than a year; *and that my cough, the severity of which made me a frequent object of sympathy, has disappeared.*

"In short, my experience under your Treatment has convinced me that no future dispensatory will be complete that does not embrace the administration, by inhalation or otherwise, of your agent or its equivalent, to those who, from their vocation or other cause, are, as I was, unable to assimilate enough of some vital element to maintain their systems in healthful vigor.

"Thanking you for renewed health, strength, and the hope of years of comfortable life, I remain

"Your grateful friend,

"WILLIAM D. KELLEY."

Testimony of T. S. Arthur, the well-known American author, who, after many years of invalidism, was restored to full health by the use of this New Treatment:

OFFICE OF *Arthur's Home Magazine.*
PHILADELPHIA, 227 S. Sixth St., Jan. 1st, 1878.

"DRS. STARKEY & PALEN: *Dear Sirs:*—I do not think I can say anything stronger in favor of your Oxygen Treatment than I have already said. Your Dr. Starkey knows how run down, enervated, and exhausted I had become, and with what reluctance and lack of faith I at last yielded to his friendly efforts to induce me to try the new agent of cure which had come into his hands.

"At that time, as I then told him, I had laid down all earnest literary work and never expected to take it up again. My friends gave me but a short lease of life. But within six months my pen was resumed, and before the year closed, I had completed one of my largest and most earnestly written books, closing the last page without any of the old sense of exhaustion. Since then, there has scarcely been a day in which I have not been hard at work in my study from three to five hours, and all this without any return of the weak and tired feeling from which I had suffered for so many years.

"The constant remark I now hear on meeting those who have long known me, is, 'How well you are looking!' And yet in a few months I shall enter my seventieth year. I am at the time of life when most men find themselves laid on the shelf and where I believed that I was about being laid some seven years ago, when I began to use this Oxygen Treatment; but now I have a sense of vitality of which I once knew nothing, and feel as if there were yet in me at least ten good working years.

"For all this I consider myself indebted to your COMPOUND OXYGEN TREATMENT.

"Yours, etc.,

"T. S. ARTHUR."

---

Two years later, Mr. Arthur says:

"The benefit received from the use of 'Compound Oxygen' has been permanent. The testimonial I gave you voluntarily, two years ago, is as true to-day, in regard to my general health, as when it was given."

And still later, June 10th, 1883, he says:

"I am still in the enjoyment of good health, which I attribute to the occasional use of Compound Oxygen."

The expense of the apparatus to prepare the gas, the time and trouble necessary to make it on a small scale for office practice, have prevented many physicians from recommending the Oxygen Treatment and prescribing it to their patients who would have otherwise been most happy to avail themselves of its benefits, and the cost of visiting Philadelphia, or some other large city, where the real Oxygen gas could be obtained, has placed the remedy beyond the reach of any but the wealthy or well-to-do classes. It has also encouraged adventurers to send out from some of the large cities various preparations under the name of Oxygenated-air, or some other title, some of which are unquestionably spurious impositions, whose only merit is that they are, generally speaking, perfectly harmless, but are incapable of producing the peculiar exhilaration and beneficial results of the genuine medicated Oxygen Inhalations.

It is now possible for any physician to procure five hundred gallons of the purest compound Oxygen Treatment for his office at an expense or only $50.00, including all the outfit for its exhibition. The usual dose being about two or three gallons per day, the cylinder would supply a number of patients for several months, and bring the price within the reach of all who are in need of this great restorative. The advertising department of the next number of this journal will give all the facts regarding the outfit for home treatment as well as to physicians, and the next article will contain a list of adjuncts to the Oxygen Treatment.

